# Supplementary material for: Short-Term Effects of Brolucizumab in the Treatment of Wet Age-Related Macular Degeneration or Polypoidal Choroidopathy Refractory to Previous Anti-Vascular Endothelial Growth Factor Therapy
Source: Medicina (Kaunas). 2022 Nov 22;58(12):1703. doi: 10.3390/medicina58121703 (PMC9784980; doi:10.3390/medicina58121703)
Supplement: Supplementary file 1 [file medicina-58-01703-s001.zip › medicina-1950014-supplementary.pdf]

Table S1. Summary of patients undergoing one injection of Brolucizumab.

[-, complications not applicable to this case; CFT, central foveal thickness (um); N, no; N/A, not applicable; PED, pigment epithelial detachment; PCV, polypoidal choroidal vasculopathy; SRF, subretinal fluid; VA, visual acuity; VEGF, vascular-endothelial growth factor; wAMD, wet age related macular degeneration]

[a]*Baseline defined as the time immediantly prior to the first injection of brolucizumab*

[b]*Improvement defined as the reduction or resolution of the indicated complication seen on OCT*

**Case no.**	**1**	**2**	**3**	**4**	**5**	**6**	**7**	**8**	**9**	**10**
**Type of patient (PCV or wAMD)**	wAMD	PCV	wAMD	wAMD	wAMD	wAMD	PCV	PCV	PCV	PCV
**Eye affected (OS or OD)**	OS	OD	OS	OD	OS	OD	OD	OD	OS	OD
**Age at treatment (years)**	70	69	76	76	63	57	67	66	52	80
**Eyes naive to anti-vegf treatment (y/n)**	N	N	N	N	N	N	N	N	N	N
**Therapy Status (n= preceding anti-vegf injections)**	8	33	17	16	29	6	11	2	16	5
**Time between last anti-vegf and Brolucizumab injection (days)**	60	30	60	90	30	30	30	30	30	90
**Baseline VA**	0.2	0.1	0.7	0.2	0.04	0.4	0.5	0.5	0.3	CF
**CRT baseline**[a] (μm)	223	337	306	234	312	344	371	347	201	1004
**SRF at baseline (y/n)**	y	y	y	y	y	y	n	y	n	n
**PED height at baseline (**μm)	N/A	140	128	384	206	454	394	837	76	439
**PED width at baseline (**μm)	N/A	1121	1036	3755	3816	75	2428	4167	1755	2448
**PED at baseline (y/n)**	n	y	y	y	y	y	y	y	y	y
**Number of Brolucizumab injection**	1	1	1	1	1	1	1	1	1	1
**CRT at 1week post-injection**	202	349	280	231	217	277	321	291	194	808
**VA at 1week post-injection**	N/A	0.3	0.5	N/a	0.1	0.1	0.3	0.4	0.4	N/A
**PED height at 1 week post injection (**μm)	N/A	97	133	344	194	421	363	744	49	2536
**PED width at 1 week post injection (** μm)	N/A	572	1058	3815	3408	67	2222	4312	1529	455
**CRT at 5 weeks post-injection** (μm)	204	329	266	N/a	221	303	288	297	191	559
**VA at 5 weeks post-injection**	0.2	0.4	0.7	N/A	0.3	0.6	N/A	0.5	0.6	N/A
**PED height at 5 week post injection (**μm)	N/A	78	163	N/A	146	504	382	764	65	2458
**PED width at 5 weeks post injection (** μm)	N/A	490	846	N/A	3354	76	2505	4333	2974	523
**Improvement**[b] **of SRF at 5 weeks post-injection (y/n/-)**	y	y	y	y	y	y	-	y	-	-

**Improvement of PED at 5 weeks post-injection (y/n/-)**	-	y	n	n	n	n	n	n	y	n

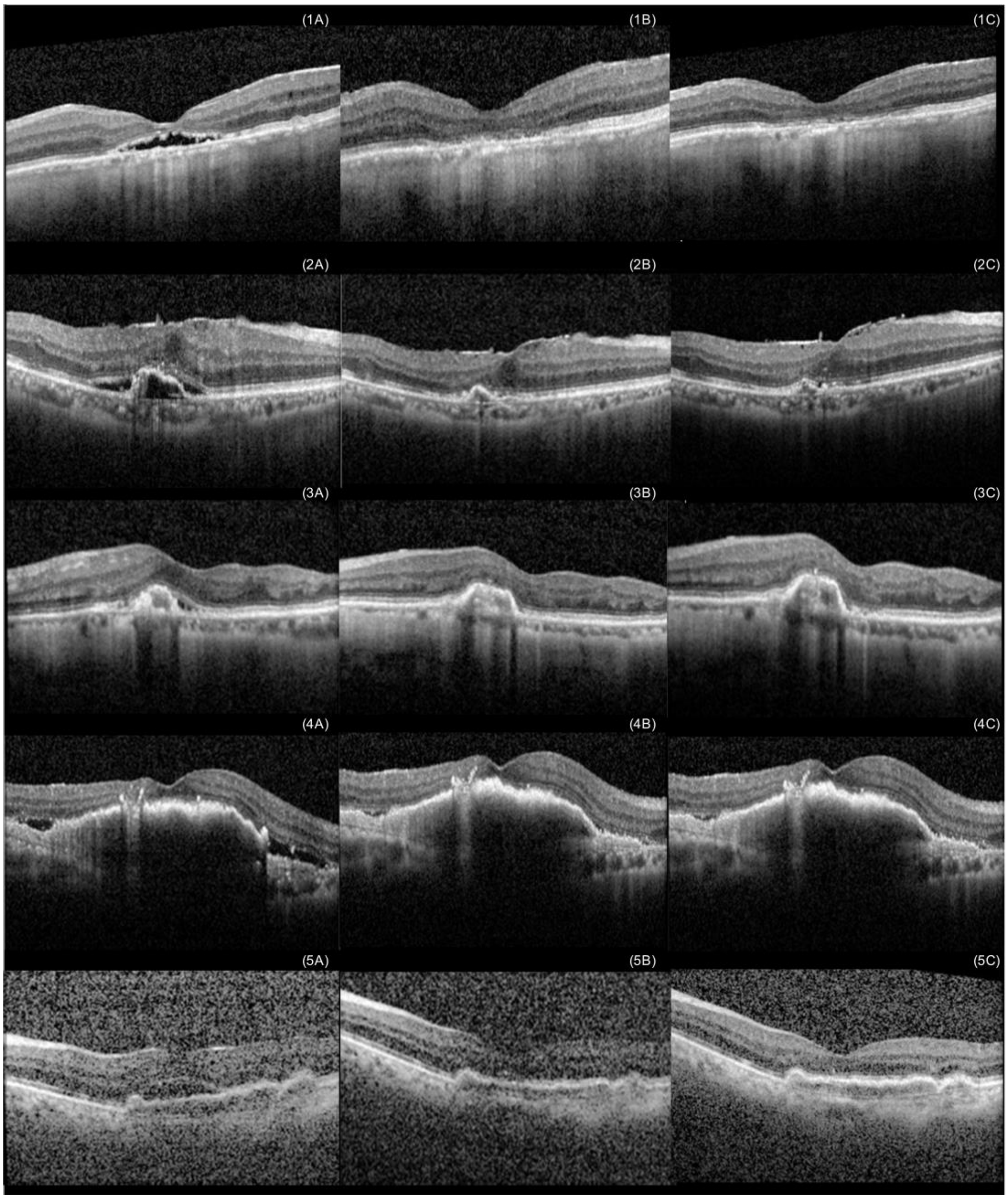

Figure S1. OCT scans for five eyes from case 1-5: (A) Baseline OCT scan before intravitreal brolucizumab treatment; (B) OCT scan 1 week after intravitreal brolucizumab treatment; (C) OCT scan 5 weeks after intravitreal brolucizumab treatment

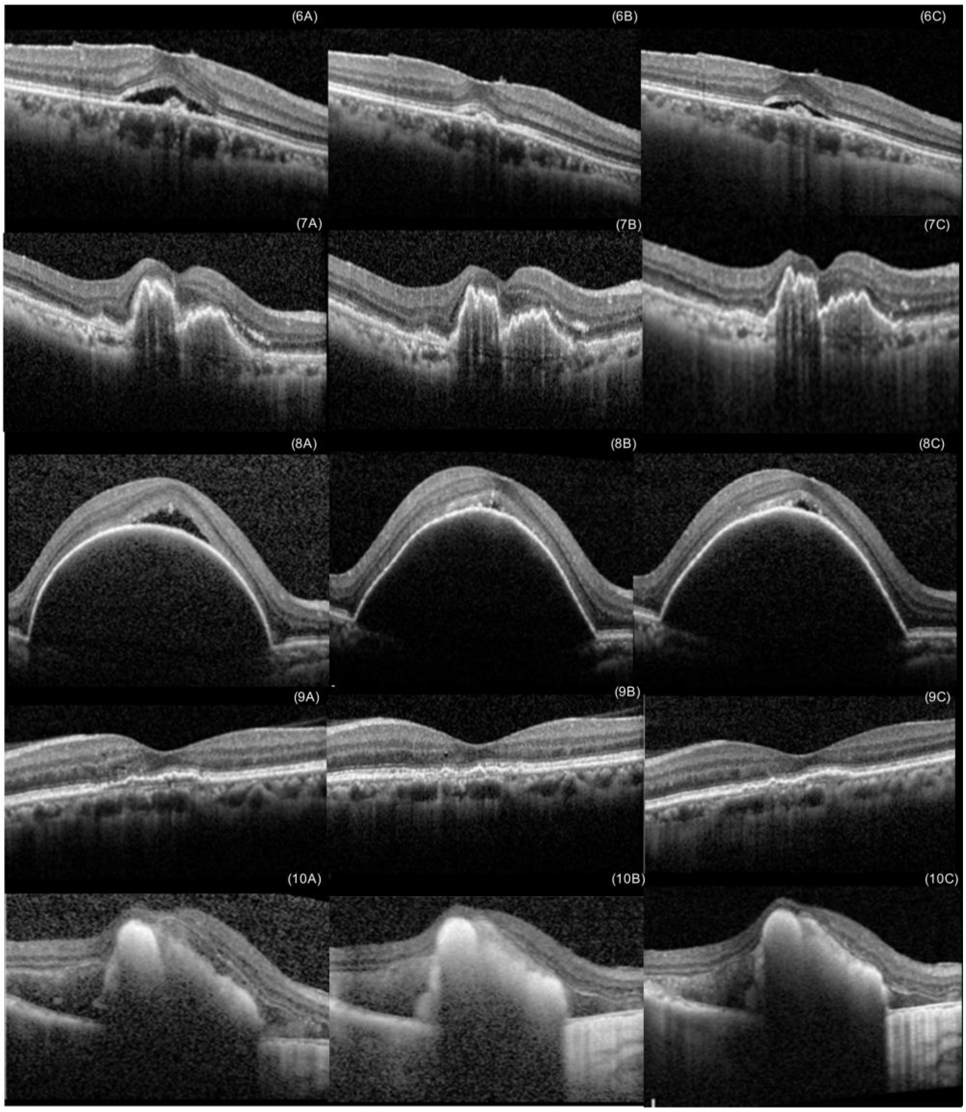

Figure S2. OCT scans for five eyes from case 6-10: (A) Baseline OCT scan before intravitreal brolucizumab treatment; (B) OCT scan 1 week after intravitreal brolucizumab treatment; (C) OCT scan 5 weeks after intravitreal brolucizumab treatment
